# The dimensions of responsiveness of a health system: a Taiwanese perspective

**DOI:** 10.1186/1471-2458-6-72

**Published:** 2006-03-17

**Authors:** Chih-Cheng Hsu, Likwang Chen, Yu-Whuei Hu, Winnie Yip, Chen-Chun Shu

**Affiliations:** 1 Center for Health Policy Research and Development, National Health Research Institutes, No.35 Keyan Road, Zhunan Town, Miaoli County 350, Taiwan; 2 Department of Economics, National Dong Hwa University, Hua-Lien 950, Taiwan; 3 School of Public Health, Harvard University, 124 Mount Auburn Street, 410S, Cambridge, MA 02138, USA

## Abstract

**Background:**

Responsiveness is an indicator used to measure how well a health system performs relative to non-health aspects. This study assessed whether seven dimensions proposed by the World Health Organization (WHO) to measure responsiveness (dignity, autonomy, confidentiality, prompt attention, social support, basic amenities, and choices of providers) are applicable in evaluating the health system of Taiwan.

**Methods:**

A key informant survey and focus group research were used in this study. The translated WHO proposed questionnaire was sent to 205 nominated key informants by mail, and 132 (64.4%) were returned. We used principal component analysis to extract factors. Linear regression analysis was used to assess the relationship between the total score and the extracted factors. A qualitative content analysis was also carried out in focus group research.

**Results:**

Principal component analysis produced five factors (respect, access, confidentiality, basic amenities, and social support) that explained 63.5% of the total variances. These five factors demonstrated acceptable internal consistency and four of them (except social support) were significantly correlated with the total responsiveness score. The focus group interviews revealed health providers' communication ability and medical ethics were also highly appraised by Taiwanese.

**Conclusion:**

When the performance of a health system is to be evaluated, elements of responsiveness proposed by WHO may have to be tailored to fit different cultural backgrounds. Four key features illustrate the uniqueness of Taiwanese perspectives: the idea of autonomy may not be conceptualized, prompt attention and choice of providers are on the same track, social support during care is trivially correlated to the total responsiveness score, and accountability of health providers is deemed essential to a health system.

## Background

Apart from the inherent goal of health promotion for the population, a responsive health system should commit itself to meet the psychological need of the people it serves and to undertake an examination of what people care about when they interact with the health system [1]. Improving these non-health functions of a health system is important because it is an inalienable component to increasing people's well-being, that being a universal and ultimate mission of a health system. Appropriately, the concept of responsiveness was introduced and a related questionnaire was also designed by the World Health Organization (WHO) to measure how well a health system meets the legitimate expectations of the population for the non-health enhancing aspects [2,3]. To emphasize its significance, the World Health Report 2000 labeled assurance of responsiveness as one of three intrinsic standards (in addition to health improvement and financial fairness) to evaluate health system performances [4].

Through the extensive processes of literature review and panel discussions, two elements were defined by WHO to measure the concept of responsiveness: respect for persons and client orientation [5]. Respect for persons, intending to capture ethical aspects of the interaction of individuals with the health system, includes three sub-elements: dignity, autonomy and confidentiality [6-8]. On the other hand, client orientation (which mainly gauges the components of consumer satisfaction) has four sub-elements: prompt attention, quality of basic amenities, access to social supports for hospitalized individuals, and choice of health providers [9,10]. Using these seven sub-elements as the framework, WHO developed a structured questionnaire to measure responsiveness [11]. In 1999, WHO conducted key-informant surveys in 35 countries to collect data on responsiveness and then used regression models to assess responsive level for the other nations that did not implement such a survey [11,12]. Finally, WHO published a global ranking of responsiveness in the World Health Report 2000.

For the intervening years since its publication, the report has generated much discussion and criticism. Some critics questioned the validity in using a single composite score to compare global health systems [13,14]. Others argued that using the prevailing "Western wisdom" of key informants (experts) to assess responsiveness might be less accurate than the judgments by patients themselves [15,16]. Blendon analyzed data from 17 European countries to demonstrate significant differences between the national ranking of WHO defined responsiveness and the satisfaction of the general public who actually experienced these health systems [17]. The report's publication also spawned contentious debates on the appropriateness of undertaking international comparisons of health system performance, given that the platforms of health systems may be quite diverse in societies with different cultural, economical or political backgrounds [18,19].

Despite these issues and debates, there has not been, to our knowledge, any empirical research undertaken studying the suitability of the seven sub-elements used by WHO as the national standard to define responsiveness for a health system. The main objective of this study, then, was to assess if the proposed seven sub-elements are applicable in Asian countries such as Taiwan. If not, we would seek to determine what components should be reorganized or what other constituents should be added in order to formulate a sounder indicator for the evaluation of responsiveness in Taiwan.

## Methods

### 1. Key informant survey

#### Questionnaire development

The questionnaire used in this study was translated from the WHO designed questionnaire for the key informant survey on responsiveness. To increase content validity, a bilingual (English-Chinese) scholar with medicine and public health background was asked to do back translation and six other public health scholars were then requested to compare the translated and original versions. If significant differences existed, a consensus needed to be reached among the six before finalizing the translated Chinese-version of the questionnaire. The main part of the questionnaire included a total score and seven conceptual sub-elements (with various amount of questions in each sub-element) to evaluate responsiveness of the health system in Taiwan [11].

#### Key informants' selection and data collection

Following the WHO documented protocol [11], 46 focal persons were identified through literature search and researchers' selection. These individuals had published papers on patient satisfaction or evaluation for health systems, were in charge of health-related agencies, or occupied a research position in health-policy-related research institutes. These focal persons were asked to nominate key informants (a minimum of three key informants was requested of each focal person). A short letter was mailed to the focal persons detailing how the key informants were selected and how the survey was to be conducted. The key informants were selected from a wide range of backgrounds including hospital clinicians, university researchers, social workers in social security departments, and officials in health or non-health related agencies. The main qualification for the key informants was familiarity with the health system of Taiwan. In total, 205 key informants were nominated through this process.

The Chinese-version questionnaire was then sent to the nominated key informants by mail in October 2003 and two follow-up reminders were delivered to non-respondents in a two-week interval. One hundred and thirty two completed questionnaires were received before the survey closed in the end of December 2003, representing a response rate of 64.4%.

#### Data analysis

The raw data was standardized to Z score to combine two types of measurements in the questionnaire, which included 28 sub-element questions, using four-pointed Likert-type scales, and seven grading questions using a rating score scaled from 0 to 10. Responses to the 35-item questionnaire were then subjected to a principal component analysis using ones as prior communality estimates. The principal axis method was used to extract factors, followed by a Promax (oblique) rotation. Any factors that accounted for at least 5% of the total variance were retained. A questionnaire item was determined to load on a given factor if its factor loading was 0.4 or greater for that factor and was less than 0.4 for the others. Cronbach alpha was used to test the internal consistency and reliability among items loaded in the same factor. Finally, a linear regression analysis was used to delineate relationship between the total responsiveness score and the extracted factors (the Taiwan model). The regression analysis was conducted using the least-squares method and three basic demographics (gender, age and employer status) of participants were also adjusted in the regression model. All statistical analyses were performed using SAS 8.01 software (SAS Institute Inc., Cary, NC, USA).

### 2. Focus group research

#### Focus group procedures

To explore ideas from the general public, focus group research was conducted. Three focus group studies were held during December 2003, each consisting of 5–12 Taipei citizens. Twenty-five people (12 men, 13 women) participated. Two groups were made up of community residents and one group comprised representatives of patient groups. Most of the participants (76%) were middle aged (median: 52 years and range: 45–65 years). Each person participated in only one group. Focus group interviews lasted for about two hours each and they were all held in a study room of National Taiwan University. Participants were offered a lunchbox and refreshments, and in the end of the session each was compensated about $20 (600 Taiwanese dollars).

Prior to the interview, participants received a letter describing the study purposes and interview agenda. The research investigator, acting as a group facilitator, opened each session with a brief introduction of the agenda, distributed and read aloud consent forms, then outlined the expectations for participants in the group and explained that participants could withdraw from the discussion at any time. The need for respect among participants and confidentiality was also stressed. The same outline of questioning to guide the discussion was used in each group. With this outline, however, we also allowed flexibility in order to collect issues most important to participants. Three primary topics in the outline were to describe (1) individual good experiences of visiting a physician; (2) individual bad experiences of visiting a physician; and, (3) characteristics that a responsive health system is supposed to have. During the last 30 minutes of each session, participants were also asked to comment on seven sub-elements of responsiveness defined by WHO.

#### Focus group analysis

Audiotapes were transcribed following each session. During the process of transcription, personal names and identifying details were removed. Full transcripts from all sessions were then examined by three researchers. The researchers read each transcript independently and reorganized important potential answers to the theme question: "In Taiwanese perspectives, what are the essential characteristics a responsive health system should be qualified with?" Areas of disagreement between researchers were compared and discussed until consensus was reached on each issue.

## Results

Table 1 summarizes the basic demographic characteristics of the survey respondents. About half (48.5%) of them were scholars, one-fourth were clinicians, and another one-fourth (26.6%) were from governmental agencies. Of respondents, males dominated (71.2%), and about half were aged below 50 years (45.5%), while those above 60 years accounted for 18.9%.

Table 2 displays the differences of factor structures constructed by the WHO study group (WHO model) and by this study (Taiwan model). In the Taiwan model, each of the retained five factors displayed eigenvalues greater than 1. Combined, these five factors accounted for 63.5% of the total variance. Using factor loading 0.4 as the cutoff criterion, 8, 9, 8, 8, and 4 items were found to load on the first (Respect), the second (Access), the third (Basic Amenities), the fourth (Confidentiality), and the fifth (Social Support) factors, respectively. The Cronbach alphas of five identified factors were between 0.79 and 0.89, indicating that their internal consistency was highly reliable.

The item contents of two factors (Basic Amenities and Social Support) were unchanged between the WHO and Taiwan models. The factor Autonomy defined in the WHO model no longer existed in the Taiwan model because it contributed two items to the Respect factor and also contributed the last remaining item to the Confidentiality factor of the Taiwan model. The factor Access in the Taiwan model essentially comprised the combination of the Prompt Attention and Choice of Provider factors of the WHO model. Most items of the Dignity factor of the WHO model were retained in the Respect factor of the Taiwan model, with the exception of one item related to the human rights issue, which was reallocated to the Confidentiality factor of the Taiwan model.

In Table 3, we used data collected in this study to delineate the relationship between the overall responsiveness score and five constructed factors in the Taiwan model. After adjusting for demographic variations, regression statistics of this model fit very well (R^2 ^= 0.70). Except for Social Support, the other four factors (Respect, Access, Confidentiality, and Basic Amenities) identified in the Taiwan model could significantly explain the variance of the dependent variable (overall responsiveness score).

After thoroughly interactive discussions, the focus-group participants regarded dignity, prompt attention and confidentiality as three most important components a responsive health system should embody. The results of the focus group interviews also indicated that medical ethics of health providers, and communication between patients and physicians were two additional areas that are priorities for Taiwanese citizens in judging the responsiveness of a health system.

Medical ethics (e.g., providers' honest behaviors and treating patients without discrimination) were frequently brought up during the group interviews. The issues that were of the most concern were discrimination against the poor and the alleged access to better health care via "red envelope" bribery. Concerning communication, most participants mentioned that in a responsive health system health providers should listen to patients carefully, explain medical terms in language that is understandable, and spend more time with patients in order to answer their questions.

## Discussion

This study has several inherent limitations. First, to our knowledge, a globally accepted assessment framework for responsiveness has not been theoretically formulated; therefore, this study just represents empirical research designed to test applicability of the WHO proposed concept in an Asian country and also to explore prospective dimensions in delineating responsiveness of the health system in Taiwan. Second, although the nominated key-informants may better comprehend achievements of a health system on the elements of responsiveness, participants of the key informant survey, as frequently criticized [17], may be biased by theoretical prejudgment. The consensus of key informants may not be able to reflect opinions of the general public, the real users of a health system. Third, participants of focus groups were volunteers who may have a vested interest in health system issues. The major reason for us to conduct a key-informant survey, instead of doing a national representative survey, was to increase comparability with the WHO estimated factor structure [11]. The seven sub-elements of the WHO proposed responsiveness measure were constructed by using key-informant surveys from 35 countries. We followed the standard protocol developed by WHO [11] to conduct the key-informant survey in order to do an item-by-item comparison in principal component analysis between factors constructed in this study and in the WHO proposed model. While bearing in mind the possible limitation of a key-informant survey, we conducted this study to do a model-building exploration. To fulfill this purpose, we also held three focus-group interviews to better understand, from the user's perspectives, embedded meanings behind the factor structure extracted in this study and also probe for new dimensions for the Taiwanese framework of responsiveness.

This Taiwanese empirical study indicates seven WHO defined sub-elements may have to be reorganized to fit Taiwanese society. Using 0.4 as the factor loading cutoff point to determine whether or not the item should be considered a good factor indicator, two original sub-element scores (Dignity and Autonomy) loaded onto two factors (Respect and Confidentiality), rather than being retained in a unique factor. On the other hand, for the newly defined factors, three out of five contained more than one original sub-element scores (Table 2).

In the element "respect for persons", the sub-element Autonomy seems not conceptualized by Taiwanese respondents as a unique domain, of which the items related to alternative treatment options (Q6 and Q7 in Table 2) were categorized into the sub-element Respect, while the item related to patient consent (Q8) was categorized into the sub-element Confidentiality. Although patient rights have recently been advocated in Taiwan, it is a tradition that the opinions of a patient's key family members (e.g., father, husband, or the oldest brother) usually determine the patient's treatment. In focus-group discussions, the participants frequently expressed the same experience: when the diagnosis of a cancer is confirmed by the pathologist, it is usually a practice routine for a physician in Taiwan to obtain authorization for treatment from the patient's key family members before informing the patient of his/her true condition. Generally speaking, this study shows less evidence of a conceptual difference between dignity and autonomy among Taiwan respondents than was found in the WHO study. It may stem from Chinese paternalistic culture that the concept of patient autonomy is not currently deemed as the "legitimate expectation" to a health system for the majority of Taiwanese people.

For the element "client orientation", the items designated to measure "prompt attention" and "choice of health providers" perfectly merged (so we named it "Access" in Table 2), indicating that unrestricted accessibility of the health system in Taiwan may pave a common trait for these two sub-elements. Prompt attention (mainly implying minimization of waiting time and waiting list) and free choice of health providers are two major achievements of national health insurance (NHI) in Taiwan. This Taiwanese compulsory health insurance, initiated in 1995, covered 97.1% of total population in 2002 [20]. Under this comprehensive coverage, there is no restriction for patients to choose physicians or medical institutes. Moreover, no referral is necessary from gate-keepers (e.g., family physicians) to visit specialists. Patients can also see any physician they prefer without incurring an extra charge. As a result, choice of health providers is not a barrier when accessing Taiwan's health services. On the other hand, the main payment mechanism in NHI is a "fee for service," which provides an incentive for physicians and medical institutes to enroll as many patients as their capacities enable; thus, in Taiwan the problems of long waiting time and waiting list are usually resolved by the system itself.

The results of the focus group discussions emphasized patient-physician communication and medical ethics to exert profound influence on the responsiveness of a health system. The communication component has already been adopted in WHO household survey for responsiveness [21]. It indicates a global consensus in improving patient-physician communication to raise the responsiveness level of a health system. On the other hand, the Taiwanese focus group participants particularly underscored the oriental viewpoints of the fundamental value of responsiveness in relation to high-standard medical ethics to treat patients fairly and empathetically. In building a measurement index for responsiveness in Taiwan, it could be suggested that the factor Communication be added to the element "client orientation," and the factor Medical Ethics be appended to the element "respect for person."

Table 3 reveals that the re-organized five sub-elements (Taiwan model) could explain 70% variability of the overall responsiveness score, the respondents' general impression to the non-health enhancing performance of the health system in Taiwan. This satisfactory result means that an equally accurate representation of responsiveness in Taiwan can be made using a revised WHO composite index (five sub-element scores) identified in this empirical study. Accompanied with two aforementioned components suggested by focus-group participants, we may thus propose a modified structure to define dimensions of responsiveness from Taiwanese perspectives. The element "respect for persons" should consist of three important factors (Respect, Confidentiality and Medical Ethics). However, the element "client orientation" should include four essential components (Access, Communication, Social Support and Basic Amenities). Of course, this proposed model requires further confirmation to validate its appropriateness in evaluating responsiveness in Taiwan. A related questionnaire has been revised and developed accordingly, and a national representative survey is also planned to carry out to solicit user view on responsiveness.

## Conclusion

When we hypothesize new perspectives of responsiveness based on this empirical study, we do not intend to declare such a measure as a universal standard. Rather, the implication of the findings asks for the recognition of the value of culture-specific aspects from different health systems when we conduct an international comparative research on quality of health care. As Groenewegen and colleagues demonstrated in their study [22], health care users in different countries usually have different ranking regards to the importance of various aspects of health care. We have to acknowledge this variation and respect both the actual experiences of people with different aspects of their health systems and the values they attach to these aspects. When the experienced responsiveness is compared internationally, the values that people attach to different aspects of health system should be estimated and hopefully the responsiveness could then be weighted by these culturally determined values.

## Competing interests

The author(s) declare that they have no competing interests.

## Authors' contributions

CCH participated in concept development, statistical analysis, and paper writing. LC and YWH both participated in questionnaire development and focus group discussion. WY participated in concept development and study design. CCS participated in questionnaire development and statistical analysis.

## Pre-publication history

The pre-publication history for this paper can be accessed here:



## Figures and Tables

**Table 1:** The demographic characteristics of surveyed key informants.

	**N**	**(%)**
Total	132	(100.0)
Employment status		
Health Related Governmental Agencies*	20	(15.2)
Other Governmental Agencies^†^	15	(11.4)
Universities and Research Institutes	64	(48.5)
Hospitals and Medical Organizations	33	(25.0)
Gender		
Male	94	(71.2)
Female	38	(28.8)
Age		
20–49	60	(45.5)
50–59	47	(35.6)
60+	25	(18.9)

* Department of Health, Bureau of National Health Insurance or other local governmental health agencies

^† ^Governmental agencies such as Ministry of Finance and Council for Economic Planning and Development

**Table 2:** Principal component analysis to identify components of responsiveness in Taiwan.

**Components of Responsiveness**		**(Taiwan model)**				
**(WHO model)**		**Respect**	**Access**	**Amenities**	**Confidentiality**	**Social Support**

	Eigenvalue	10.675	4.518	3.416	1.949	1.655
	Cronbach's alpha*	0.891	0.811	0.856	0.876	0.788
**Dignity**						
1	Treated with respect	**0.530**	0.149	0.030	0.251	0.032
2	Common human rights	0.351	0.033	0.010	**0.483**	-0.045
3	Free to discuss concerns	**0.816**	-0.027	0.063	-0.041	0.081
4	Encouraged to ask questions	**0.831**	0.012	0.115	-0.046	0.013
5	Respect privacy in physical examination	**0.661**	-0.099	-0.059	0.325	0.033
5A	Overall score	**0.465**	0.132	0.122	**0.445**	-0.128
**Autonomy**						
6	Provided with information	**0.830**	-0.016	-0.094	0.046	0.021
7	Consult on preferences	**0.835**	-0.083	0.033	0.002	0.059
8	Patient consent	0.257	0.096	-0.162	**0.644**	-0.131
8A	Overall score	**0.529**	0.042	0.037	**0.437**	-0.095
**Confidentiality**						
9	Protect confidentiality	0.369	-0.011	-0.106	**0.624**	-0.050
10	Confidentiality of patient information	0.090	-0.131	0.004	**0.881**	0.087
11	Confidentiality of medical records	-0.077	-0.093	0.059	**0.816**	0.158
11A	Overall score	0.077	0.042	0.063	**0.831**	0.049
**Prompt attention**						
12	Geographic access	-0.065	**0.739**	-0.106	-0.093	-0.037
13	Fast access to emergency care	-0.035	**0.711**	-0.130	0.059	-0.004
14	Reasonable waiting time	-0.042	**0.603**	0.352	-0.034	-0.166
15	Reasonable waiting time for non-emergency surgery	-0.091	**0.756**	-0.016	0.032	0.087
15A	Overall score	-0.188	**0.836**	0.029	0.253	-0.005
**Social support**						
16	Allow visitors	0.088	0.023	0.010	-0.032	**0.829**
17	Take care of personal needs	0.146	0.107	0.001	-0.087	**0.814**
18	Practice religious acts	-0.095	-0.062	0.036	0.151	**0.735**
18A	Overall score	-0.199	0.355	0.045	0.289	**0.541**
**Basic amenities**						
19	Cleanliness of unit	0.105	-0.160	**0.752**	-0.060	0.139
20	Maintenance of building	-0.115	-0.167	**0.801**	0.132	0.081
21	Adequacy of furniture	0.030	-0.164	**0.720**	0.043	-0.072
22	Nutrition and edibility	0.029	0.389	**0.466**	-0.016	-0.231
23	Clean water	-0.002	0.207	**0.666**	-0.111	0.040
24	Cleanliness of toilets	0.069	-0.093	**0.794**	-0.099	0.095
25	Cleanliness of linen	0.125	0.171	**0.656**	-0.094	-0.064
25A	Overall score	-0.114	0.079	**0.811**	0.219	-0.018
**Choice of providers**						
26	Choice of provider at health care unit	0.231	**0.630**	0.074	-0.134	-0.100
27	Choice between units	0.147	**0.552**	0.009	-0.093	0.391
28	Choose to see specialist	0.122	**0.666**	-0.116	-0.185	0.222
28A	Overall score	0.072	**0.783**	0.015	0.080	0.122

* Correlation of selected items (factor loading>0.4) in each factor

**Table 3:** The relationship between total responsiveness score and the five extracted factors^†^

**Taiwan Model**	**Parameter (Std Err)**
Intercept	0.574 (0.304)
Respect	0.203 (0.079)*
Confidentiality	0.229 (0.082)**
Access	0.351 (0.066)***
Social Support	0.075 (0.060)
Basic Amenities	0.313 (0.064)***
R^2^	0.700
F-value	27.37
P-value	<0.0001

Note. *<0.05 **<0.01 ***<0.001

^† ^The relationship is assessed by using a linear regression analysis: the dependent variable is the total responsiveness score and the demographic variables (gender, age and employer status) have been adjusted
